# “Edible Beauty”: The Evolution of Environmentally Friendly Cosmetics and Packaging

**DOI:** 10.3390/antiox13060742

**Published:** 2024-06-19

**Authors:** Irene Dini

**Affiliations:** Department of Pharmacy, University of Naples Federico II, Via Domenico Montesano 49, 80131 Napoli, Italy; irdini@unina.it

**Keywords:** nutricosmetic, food supplements, edible packaging, antioxidant-based edible packaging, circular economy, green economy, green cosmetics

## Abstract

The cosmetics industry plays a significant role in the global economy and consumer lifestyles. Its dynamic and adaptable characteristics make it a key player worldwide. The cosmetics industry generates enormous profits globally, injecting billions of dollars into the world’s economy each year. The industry’s marketing efforts, product launches, and trends influence consumer behavior and perceptions of beauty, contributing to cultural dialogues and societal norms. This study, conducted with a rigorous bibliometric and systematic literature review, offers a comprehensive overview of recent progress in edible cosmetics. The “skincare you can eat” is an innovative branch of cosmetics that employs food co-products and by-products to create edible skincare and hair products and edible packaging materials to advance human well-being and sustainability while honoring the ecological boundaries of our planet. Nutrients and antioxidants derived from organic waste are used in cosmetics and packaging. Some doubts remain about the capacity of edible packaging to be attractive to consumers and offer a reasonable shelf life for cosmetics, and also about safety. It is desirable for the authorities to guarantee consumer health through carefully regulating labeling requirements and good manufacturing practices for cosmetics and edible packaging.

## 1. Introduction

The cosmetics industry has an enormous influence and economic impact on a global scale. It is a critical and rapidly expanding sector estimated to reach around USD 736.80 billion by 2028 [1]. Within the cosmetics market, skincare, haircare, make-up, perfumes, toiletries, deodorants, and oral cosmetics are key product categories that meet consumer demands for personal care products. Skincare emerged as the leading segment in 2023, capturing substantial consumer spending and market share. The cosmetics industry has seen significant advancements in adopting innovative technologies and practices to reduce environmental impact throughout product lifecycles, from sourcing raw materials to manufacturing, packaging, distribution, and disposal [1].

A significant trend in recent years has been the growing convergence of food and cosmetics in the beauty industry. This intersection offers exciting possibilities, such as foodie cosmetics that are especially popular among Millennials and Generation Z [1]. Superfoods (such as licorice, pomegranate, green tea, jojoba, and cacao) [2], nutraceuticals (natural ingredients and supplements to promote overall well-being through an integrated approach that considers the body, mind, and spirit) [3], and the desire for sustainability and waste reduction (use of food waste by-products to make cosmeceuticals) [4] are increasingly present in cosmetic formulations. Companies increasingly source raw materials from sustainable and renewable sources, such as organic farms, responsibly managed forests, and fair-trade cooperatives, to reduce deforestation, protect biodiversity, and support local communities. Natural and biodegradable ingredients and processes that produce fewer pollutants and require fewer resources are employed to formulate green and eco-friendly cosmetics. Eco-friendly packaging, such as bioplastics or paper fibers, compostable materials, and recycled packaging (i.e., recycled plastic or paper fibers), minimizes plastic waste, lends more efficient transportation and storage, and promotes circularity. Antioxidants from organic waste are incorporated into cosmetics to mitigate cellular aging and integrated into packaging materials to decrease the rate of cosmetic oxidation and extend their shelf life [3].

Additionally, efforts are being made to reduce packaging sizes, optimize transportation logistics, and provide clear instructions on recycling or properly disposing of packaging. This review provides an overview of recent advancements in the recycling and upgrading of organic waste to produce edible cosmetics and edible packaging materials. The analysis explores how the cosmetics sector has aligned with the principles of the 4Rs of the circular economy (reduce, reuse, recycle, and recover) to minimize waste production, maximize resource efficiency, and promote circularity throughout the entire lifecycle of cosmetics [5]. This study aims to identify critical cosmetic sector gaps and trends and recommend future research directions and industry practices. 

## 2. Materials and Methods

### 2.1. Search Strategy

The literature review employed Preferred Reporting Items for Systematic Reviews and Meta-Analyses guidelines (PRISMA) to ensure the validity and robustness of the research findings [6]. The information sources included Scopus, WOS, Google Scholar, Google Patent, and the Statista website.

### 2.2. Inclusion Criteria

The eligibility criteria included any scientific papers (research article, review, editorial, or communication) and patents addressing cosmetics that fell under the following categories: (1) food waste recycling, (2) food cosmetics, and (3) edible cosmetic packaging.

The exclusion criteria were non-English articles and papers from non-academic sources (e.g., newspapers, internet websites, magazines, etc.). The exact search strategy included work from 2002 to 2024. The search terms were “food waste recycling”, “food cosmetics”, “edible packaging”, and “cosmetic edible film”. There were 109 revised articles.

## 3. Cosmetics

The EU Cosmetic Products Regulation No. 1223/2009 [7] regulates the sale of cosmetic products in Europe. It defines “cosmetic products” as chemical elements and their compounds and mixtures created with two or more substances projected to be employed in contact with the epidermis, hair system, lips, nails, external genital organs, teeth, and the oral cavity’s mucous membranes for cleaning, perfuming, changing appearance, correcting odors, protecting, or keeping them in good condition [7]. 

Cosmetics and personal care items include creams, emulsions, lotions, gels, and oils for skin and hair cleansing, nourishment, and make-up application. This category also contains perfumes, antiperspirants, and any substance intended for inclusion as a component of a cosmetic product.

### 3.1. Green Cosmetic

**Figure 1:**
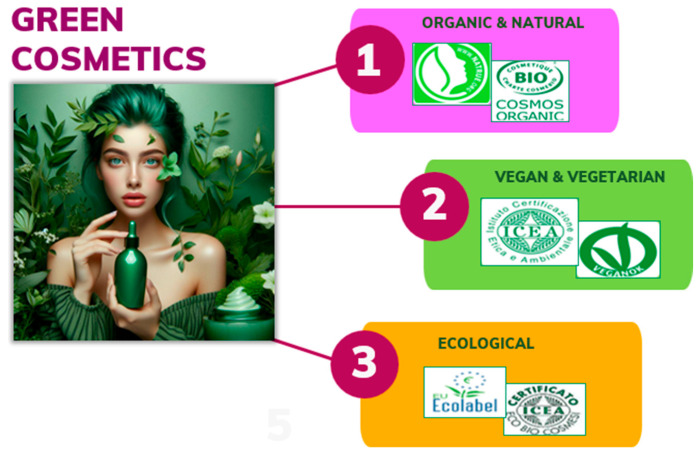
Specialized private bodies that provide “green” certifications for cosmetic products.

A precise and universally accepted definition of “green cosmetics” is somewhat elusive, as interpretations can vary among consumers, companies, and regulatory bodies [8]. A “green cosmetic” typically refers to cosmetics formulated with environmentally friendly, sustainable, and non-toxic ingredients sourced from renewable resources produced using environmentally responsible manufacturing processes, such as minimizing waste generation, reducing water consumption, and utilizing eco-friendly packaging materials [9]. Green cosmetics generally adhere to specific standards (i.e., Natrue, Cosmos, etc.) or certifications (i.e., Icea, CCPB, Ecocert, Demeter, etc.) (Figure 1), demonstrating their commitment to ethical and sustainable practices such as being cruelty-free (products that are not tested on animals at any stage of production, from ingredients to finished products), vegan (products that do not contain any animal-derived ingredients and are not tested on animals), or free from certain harmful chemicals like parabens, phthalates, and sulfates [8]. Each type of cosmetic offers consumers different choices based on their preferences for ingredients, ethics, and environmental concerns. Natural cosmetics use natural ingredients, organic cosmetics adhere to strict farming standards, vegan cosmetics exclude animal-derived ingredients, and ecological cosmetics prioritize sustainability throughout production. 

#### 3.1.1. Natural Cosmetics

Natural cosmetics are products formulated primarily with natural ingredients, such as plants, minerals, and animal products. These ingredients are minimally processed, may be sourced from organic or conventional farming practices, and are often chosen for their beneficial properties. Natural cosmetics may contain organic and non-organic ingredients and prioritize the use of natural substances rather than synthetic chemicals [9]. 

#### 3.1.2. Organic Cosmetics

Organic cosmetics are formulated with ingredients that have been grown and harvested following strict organic farming practices. These ingredients are free from synthetic pesticides, fertilizers, and genetically modified organisms (GMOs). Regulatory bodies often certify organic cosmetics to ensure compliance with organic standards and environmental sustainability [10]. The terms “100% organic”, “organic”, and “made with organic” are employed to indicate different levels of organic content in cosmetic products. “100% organic” indicates that all ingredients in the product are certified organic. “Organic” typically contains at least 95% organic ingredients. “Made with organic” implies that the product contains at least 70% organic ingredients [10]. 

#### 3.1.3. Vegan Cosmetics

Vegan cosmetics are products that do not contain any animal-derived ingredients or by-products, such as beeswax, honey, lanolin, or carmine. Vegan cosmetics are cruelty-free and are not tested on animals. While vegan cosmetics may contain natural or organic ingredients, their primary distinction is the absence of animal-derived components [11]. 

#### 3.1.4. Ecological Cosmetics

Ecological cosmetics, also known as eco-friendly or sustainable, prioritize environmental responsibility throughout their entire lifecycle, sustainably sourcing ingredients, minimizing waste, using renewable energy in production, and employing eco-friendly packaging materials. Ecological cosmetics aim to reduce environmental impact while delivering practical skincare benefits [12]. 

#### 3.1.5. Food-Recycled Materials (By-Products and Co-Products)

Organic waste is any waste made from plant and animal matter. Across the European Union, an estimated 118 to 138 million tons of bio-waste is produced annually. This significant amount of bio-waste presents both a challenge and an opportunity. With proper management, it can be transformed into valuable resources. Organic waste contains polymers (polysaccharides, protein), lipids, and bioactive compounds (such as polyphenols, flavanols, and tannins) that can be used for cosmetic film formation and to enhance the antioxidant and antimicrobial properties of edible packaging. 

Industrial agri–food systems generate many co-products and by-products. The categorization of products as by-products or co-products can vary depending on the context, industry norms, and regulatory frameworks. Some products may even qualify as both by-products and co-products concurrently, further adding to the complexity of the distinction. Nevertheless, precise definitions and context-driven interpretations are essential [13]. 

Food co-products are secondary but intentional outcomes that can be creatively or functionally utilized, while food by-products are incidental and unintentional outcomes of the production process [14]. 

Co-products, being intentionally produced and potentially having comparable value to the main product, may undergo different marketing strategies and allocation decisions to maximize their value and utilization.

By-products may be considered less valuable and could be used for animal feed, composting, or bioenergy production. By-products derived from animal processing (livestock and poultry sectors) are subdivided into three categories based on their origin and the potential risks for humans, animals, and the environment. Animal parts unsuitable for food or feed use (such as brains, bone marrow, and spinal cords) are classified as Category 1. Disposal is the sole lot of those by-products. Category 2 includes fewer risks than Category 1 but is still unsuitable for feeding. It can be used as organic fertilizer or compost. Category 3 involves no risk and is suitable for feeding animals and pets [15]. The by-products of fisheries, aquaculture, dairy, cereals, vegetables, and fruit can be used in edible cosmetics, food, nutraceuticals, and feed [16]. The European Union project FUSIONS considers food by-products to be substances or objects that can undoubtedly be used again or utilized without further processing, except for typical industrial practices, whose specific use does not lead to adverse ecological or human health impacts [17]. 

#### 3.1.6. Edible Cosmetics

**Figure 2:**
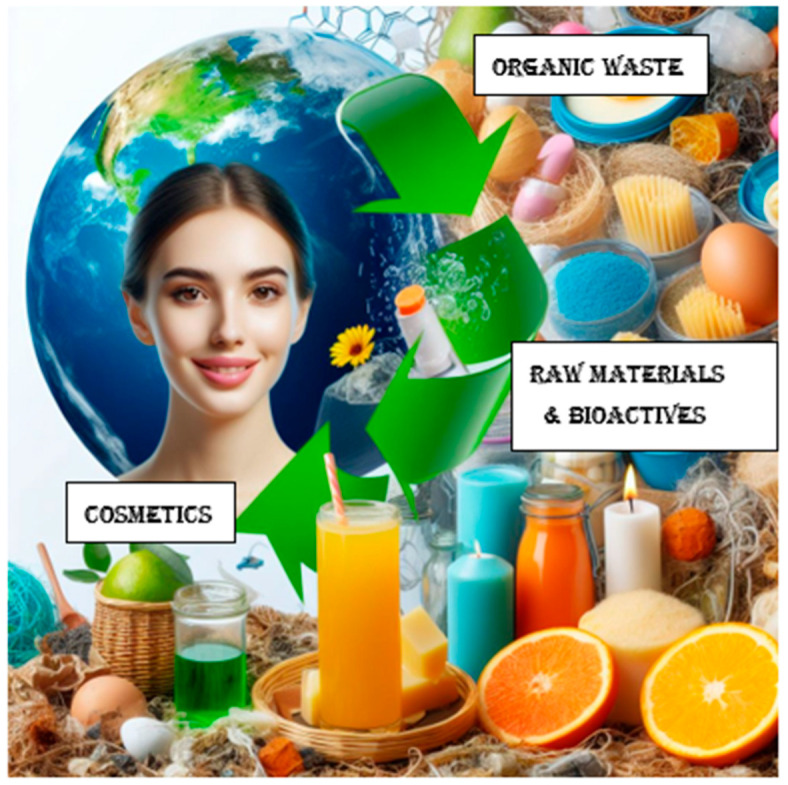
Suitable reuse of organic waste.

Edible cosmetics are cosmetics formulated exclusively with food-grade materials (Figure 2).

##### Edible Polymers

Natural polymers serve in cosmetic and personal care products as rheological modifiers, thickeners, water-soluble binders, conditioners, film-forming agents, active ingredients, sensory ingredients, moisturizers, texturizing agents, and hydrating agents (Table 1 and Table 2). 

##### Antioxidants from Organic Waste in Cosmetics

Antioxidants are substances commonly utilized to inhibit or decelerate the process of oxidation that can lead to the deterioration of cosmetics. Through chelating the catalysts, they can eradicate the originators of reactive nitrogen species (RNS) and reactive oxygen species (ROS). Alternatively, they can remove or neutralize free radicals produced during the reaction’s start or progression through contributing hydrogen atoms to these molecules. The application of antioxidants in cosmetics reduces oxidative damage, constituting a good alternative in therapy and preventing premature aging. They also provide photoprotective action and help treat sensitive or sun-stressed skin with their anti-inflammatory activity. Antioxidant compounds are also applied to improve cosmetics’ shelf life. They prevent or reduce oxidative deterioration of active constituents of the cosmetic and avoid oxidation of oily content present in the formulation [3]. In addition, phenolic compounds exhibit antibacterial and antifungal properties. The antibacterial action is attributed to their ability to damage the cytoplasmic membrane, inhibit nucleic acid synthesis, and suppress microorganisms’ energy metabolism. The antifungal properties of these substances are associated with their capability to disrupt the biosynthesis of the cell wall, interfere with the synthesis of folate metabolism, and subsequently inhibit the production of ergosterol. They also help reduce biofilm formation and enhance the membrane’s ionic permeability [4].

##### Edible Capsules or Pods

Edible capsules or pods made from seaweed extract, starches, or edible films are employed for lotions, serums, or creams. Consumers can tear open the pod and apply the product directly onto their skin (Table 3). 

##### Edible Powders or Tablets 

**Figure 3:**
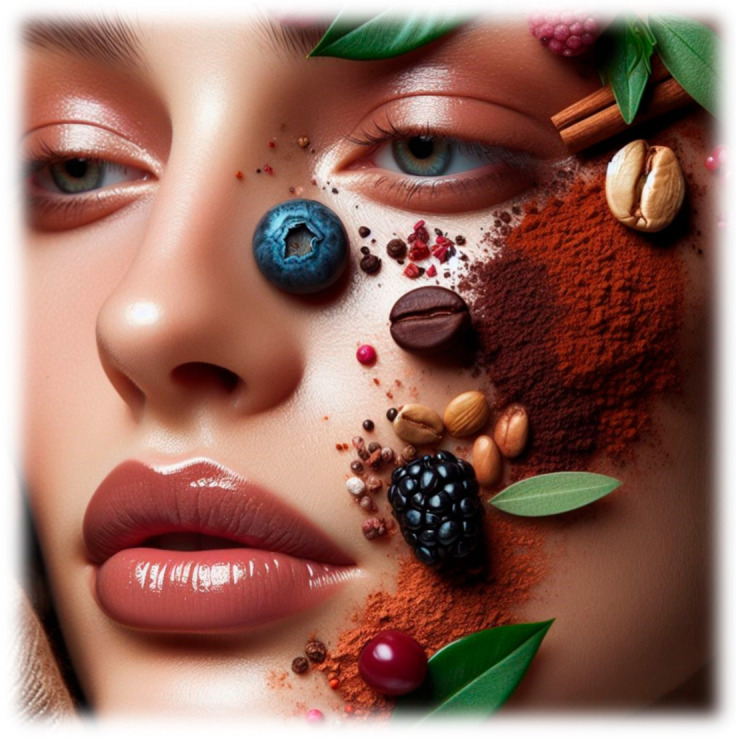
Edible powders.

Cosmetic powders like foundation, eyeshadow, or blusher are compressed into edible tablets or capsules made from rice flour or vegetable starch. These tablets can be applied to the skin using a damp sponge or brush, and the remaining residue can be ingested or washed away (Figure 3 and Table 4).

##### Edible Lip Balms or Lipsticks

**Figure 4:**
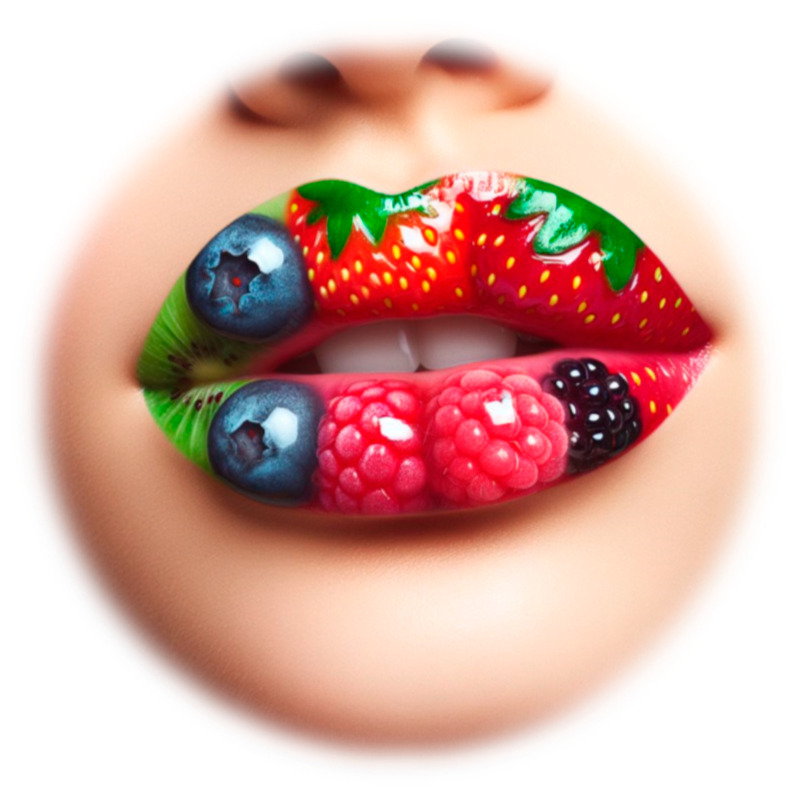
Edible lip balms or lipsticks.

Lip balms or lipsticks can be formulated using plant-based waxes, oils, and natural pigments. The packaging for these products can also be edible (e.g., a thin layer of fruit-based gelatin or agar coating the product) (Figure 4 and Table 5).

## 4. Packaging

The packaging is a barrier against physical, chemical, and biological factors that can cause product degradation. It protects cosmetics (preventing contamination, moisture, light, and air, and providing integrity during transportation, storage, and handling) and is a powerful branding and marketing tool. The packaging must comply with various regulatory requirements and standards for product safety, labeling, and environmental sustainability. The size of the cosmetics packaging market was USD 55,729.9 million in 2023. It is projected to increase to approximately USD 58,070.6 million in 2024 and grow to USD 78,612.4 million by 2032, with a compound annual growth rate (CAGR) of 3.9% from 2024 to 2032. [41]. The cosmetic packaging market continues its growth, driven by evolving consumer preferences, innovation in materials, and sustainable packaging solutions. Key drivers propelling market growth include the surge in beauty and personal care products, heightened emphasis on eco-friendly packaging, and the advent of premiumization in the cosmetics industry. Petrochemical plastics (i.e., PET (polyethylene terephthalate), PVC (polyvinylchloride), PS (polystyrene), and PA (polyamide) are usually used for cosmetic packaging since they are transparent, permeable, flexible, have good tensile strength, thermal efficiency, cost-effectiveness, and can be sterilized. However, the overuse of these materials is unsustainable and has been proven to have disastrous consequences for our planet. Plastics contribute to worldwide environmental pollution and threaten various life forms since they have a stable structure of carbon–carbon bonds [42]. Synthetic plastics not only take centuries to break down, but they also seep into the environment. It has been estimated that 8 million metric tons of plastics end up in our oceans each year [5]. Plastics disperse and gradually break down into micro- and nanoplastics when they enter the ocean. Various organisms, including marine mammals, fish, crustaceans, mollusks, zooplankton, and phytoplankton, consume these tiny particles. This ingestion can adversely affect their physiological processes [43], impacting climate change and global warming since approximately 70% of the world’s oxygen is generated through photosynthesizing marine life forms such as seaweeds and microalgae [44].

Furthermore, people ingest plastic particles by consuming land and sea-based food products, drinking water, and inhalation [45]. This scenario has increased demand for alternative packaging solutions that are renewable, recyclable, easily degradable, and require little to no disposal. Edible packaging materials, a subset of bio-based and biodegradable materials, have been researched as an alternative to traditional cosmetic packaging due to their film-formation properties.

### 4.1. Edible Packaging

**Figure 5:**
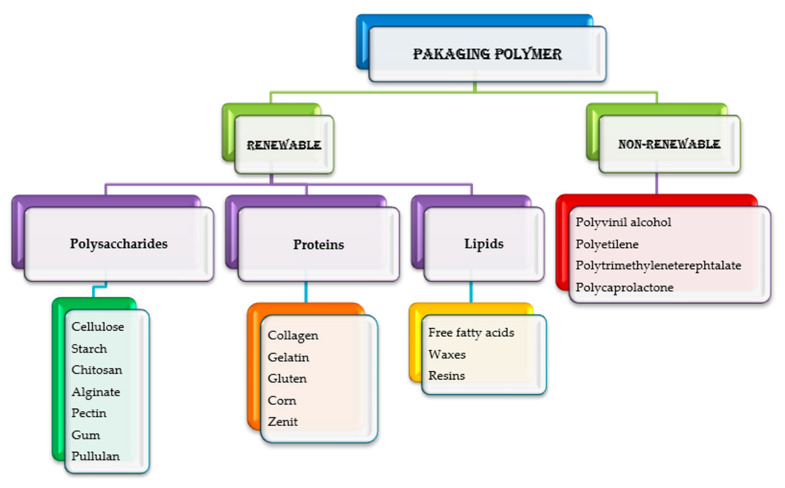
Packaging materials.

Edible packaging is an eco-friendly, safe, and functional solution that can be consumed with the product and decomposes faster than traditional packaging materials, significantly reducing the waste generated. Edible packaging provides a novel experience that can attract consumers looking for unique products, allowing brands to stand out in a crowded market with an eco-friendly image and increasing consumer engagement and loyalty. Edible packaging is based on biomolecular matrixes derived from plants, animals, or microorganisms. Common biomolecules include polysaccharides (i.e., cellulose, starch, and chitosan), proteins (i.e., casein, gelatin, and soy proteins), and lipids (i.e., paraffin, waxes, and oils) [46] (Figure 5). Biopolymers form a cohesive structure that incorporates active compounds (i.e., prebiotics, probiotics, vitamins, minerals), functional ingredients (i.e., antioxidants, antibrowning agents, and antimicrobials), and sensory enhancers (i.e., flavors, colors, and textures enhancers) [13]. Edible packaging employs edible materials (i.e., capsules, pods, film, strips, powder, tablets, lip balms, lipsticks, sachets, and pouches) to package cosmetic products, offering a sustainable alternative to reduce plastic waste and the environmental impact of traditional cosmetics packaging and promote environmental responsibility in the beauty industry.

#### 4.1.1. Edible Films or Strips

**Figure 6:**
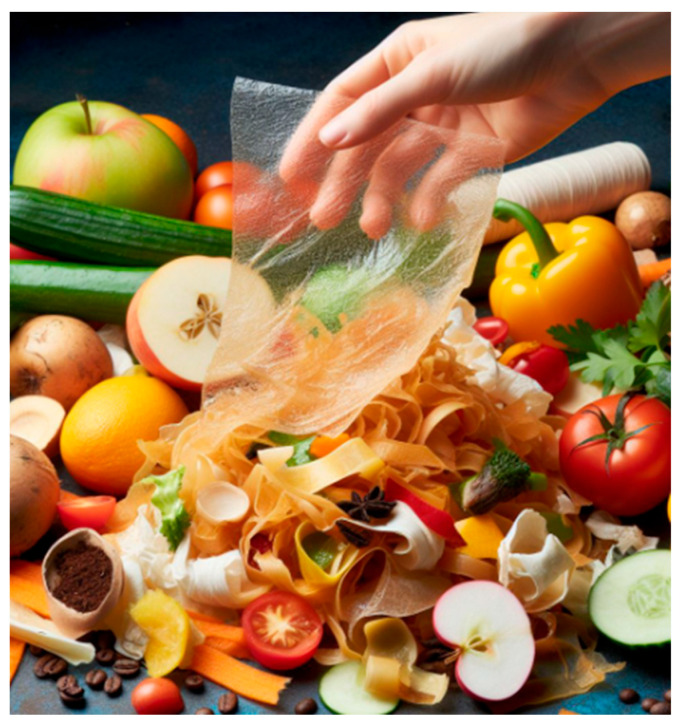
Recycling organic waste to create edible films.

The materials used to create edible films can be grouped into three primary categories based on their source and production process: polymers that are directly extracted from biomass, such as polysaccharides and proteins; polymers that are produced through traditional chemical synthesis using renewable, bio-based monomers, like polylactic acid, and polymers that are produced by microorganisms or genetically modified bacteria [47] (Figure 6). 

Films based on biopolymers are created using solutions that contain three primary components: the biopolymer, a plasticizer, and a solvent. The characteristics of the resulting film are influenced by both the inherent properties of the film components and external processing factors. Protein films offer mechanical stability, polysaccharides are used to manage oxygen and other gas transmissions, and fats are employed to minimize water transmission [48]. It is important to note that only protein films supply nitrogen during their degradation, serving as fertilizers, a benefit not provided by films that do not contain protein [49]. Polysaccharides are carbohydrate macromolecules composed of two or more monosaccharides connected with glycosidic linkages through condensation reactions, insoluble in alcohol and nonpolar solvents, typically white in color, tasteless, and seldom crystalline. Thanks to their adaptable biocompatibility and various functionalities, they stand out among edible polymers [50]. Starch, pectin, carrageenans, alginates, xanthan gum, and cellulose derivatives are edible polysaccharides used in cosmetics [51,52,53]. 

Fruit pectin [54] or agar [55] can be used for facial masks, exfoliating pads, or individual make-up wipes. These films dissolve upon contact with water, releasing the product and eliminating the need for additional packaging waste. They possess thermal characteristics, barrier attributes, and mechanical properties. Thermal analysis helps understand how a material behaves under varying rates of cooling or heating or in an inert, reducing, or oxidizing atmosphere. This understanding allows prediction of how a protein package might behave during different processing stages, such as freezing or cooking. During these stages, proteins denature, dissociate, and realign, allowing protein molecules to combine and cross-link through specific linkages. In the context of packaging materials, a critical piece of information is the glass transition temperature (Tg, the temperature range at which a glassy material transitions into a rubbery state, decreasing the Young’s modulus). 

The term “glass transition” describes the reversible shift in the physical characteristics of specific materials when they undergo a particular range of temperature changes. Biopolymer-based films exhibit brittleness at lower temperatures. However, as the temperature increases and reaches the glass transition point, these materials transform and exhibit ductility.

Above Tg, the polymeric materials are in a soft, rubbery state, which has barrier properties, while below Tg, polymers are in a glassy state with low permeability. The Tg values are also vital for determining the compression molding and extrusion temperatures. Tg generally increases with stiff chains and bonds, cross-linking between chains, bulky side groups, and the degree of crystallinity. Conversely, Tg decreases when the quantity of low-molecular-weight plasticizers increases [56]. Barrier properties are crucial in determining a product’s shelf life and/or packaging, and they are linked to the product’s requirements and final use. The primary agents studied in packaging applications are water vapor and oxygen, as they can permeate from the internal/external environment through the polymer, leading to ongoing changes in product quality and shelf life. Biopolymer-based films typically have a high inclination towards water vapor permeability (WVP), making the solubility and diffusivity of water molecules critical factors in controlling permeability within the polymeric matrix. WVP coefficients indicate the amount of water vapor that passes through a unit area of the packaging material in a given time (kg mm^−2^ s^−1^ Pa^−1^) and quantify water vapor permeability [57]. The polymers influence the mechanical properties. The tensile test measures the tension force in Pascal (MPa), the percentage of elongation at break (%), and the elastic modulus in Pascal (GPa). It provides insights into the material’s flexibility, hardness, and elongation and helps to predict how the packaging behaves during handling and storage [58]. 

#### 4.1.2. Polysaccharide-Based Edible Films

Polysaccharide-based edible films have good gas oil and lipid barrier performance but are vulnerable to humidity and have low water resistance. Cellulose, alginate, and pectin are the leading polysaccharides used. The key advantages of polysaccharides are availability, abundance, nontoxicity, thermo-processability, and low cost. Cellulose requires a chemical alteration, which involves replacing the numerous hydroxyl functions with acetate or methyl groups before being used for packaging purposes. As a result of this modification, the material becomes more straightforward to process and convert into films due to the reduction in the physical system and the quantity of hydrogen bonds. Various cellulose materials can be produced following this chemical adjustment, each with specific mechanical strength characteristics, solubility, and barrier efficiency against oxygen and lipids. The most frequently used materials, known for their excellent film-forming properties, include hydroxypropyl methylcellulose, methylcellulose, hydroxypropyl cellulose, and carboxymethyl cellulose [59]. Alginates are obtained from brown seaweeds; they have good oxygen and water barrier qualities, antioxidant properties, flexibility, and absorbency ability [60]. Edible films made from pectin are used in solution-cast or compression-molded self-standing films [61], extruded casings [62], or edible coatings [63].

#### 4.1.3. Proteins-Based Edible Films

Edible protein films are obtained from proteins in plants and animals (Table 4). The advantages of proteins over polysaccharides are their abundance, high nutritional value, remarkable ability to form films, gas barrier properties against odor, oxygen, and carbon dioxide, and mechanical properties [64]. Their flexibility is enhanced with the presence of numerous hydrophilic substances such as sorbitol and glycerin. However, changes in the moisture content of the surrounding environment can negatively impact their mechanical properties and water vapor permeability due to their natural hydrophilicity and consequent high biodegradability. Applying cross-linking, which can be either chemical or physical, can potentially improve these characteristics [65]. Collagen-based edible film is a protective layer, preventing the movement of oxygen, moisture, and solutes. It contributes to the product’s structural integrity and allows vapor permeability. Additionally, such films inhibit fat oxidation, discoloration, and microbial growth, thereby preserving the product’s sensory attributes [66]. 

Gelatin is obtained through the partial hydrolysis and heat treatment of collagen. The chemical composition of gelatin is closely related to that of collagen. Hydrolysis breaks down the natural molecular bonds between individual collagen strands into a more easily rearranged form. As a result, gelatin is a combination of single or multiple-stranded polypeptides, each with an extended left-handed helix conformation and containing between 50 and 1000 amino acids. The molecular weights can range from 3 to 200 kDa, influenced by the type of raw material used and the specific handling conditions [67]. Two gelatin varieties are typically produced: type A (from acid hydrolysis) and type B (from alkaline hydrolysis) [68]. Gelatin is rich in glycine residues, which comprise nearly one-third of its structure and are arranged in every third residue. It also contains proline and 4-hydroxyproline residues. A typical gelatin structure is -Ala-Gly-Pro-Arg-Gly-Glu-4Hyp-Gly-Pro-. The estimated composition of amino acids in gelatin can fluctuate, particularly among the minor constituents, based on the source of the raw material and the processing method used [69]. Gelatin-based films are an excellent barrier against oxygen but have a high sensitivity to moisture that decreases their thermomechanical and barrier properties. A potential solution to this issue is to combine a gelatin-based film with a moisture-resistant biodegradable polymer through a process known as laminating or coextrusion, which is commonly used in film packaging. This process optimizes multilayered structures based on the specific packaging requirements and conditions [70]. 

Zein possesses excellent film characteristics, including a water-repelling nature (despite having high levels of nonpolar amino acid groups), potential antimicrobial and antioxidant activities, adhesive film-forming capability, and exceptional resistance to both moisture and oxygen. The Food and Drug Administration (FDA) recognizes zein as a safe material for use in food systems. The disadvantages of zein-based film are its tendency to break easily and its poor processability, mechanical properties, elongation at break, and thermal properties [71]. 

Films derived from whey proteins are known for their viscosity and stretchability. They exhibit a strong barrier against oxygen and aromas thanks to their densely packed, orderly network structure and low solubility. However, due to the high content of hydrophilic amino acids in whey protein, products made solely from whey protein materials may have subpar mechanical strength and vapor-barrier properties. Plasticizers, antimicrobial agents, or antioxidants can bolster their mechanical strength and augment their functional properties. The high cost of whey proteins limits their use in this sector [72]. 

#### 4.1.4. Lipid-Based Edible Films

Lipids have a low molecular weight and polarity; therefore, they provide moisture-barrier properties but cannot be applied alone as films. Lipids commonly utilized in lipid-based edible films are mono-, di-, and tri-acylglycerols, free fatty acids, phospholipids, waxes (i.e., beeswax, candle wax, and carnauba wax), and resins (i.e., shellac resin, turpentine, and coumarin resin) (Table 6) [73]. Lipid films lack flexibility and transparency. Lipid molecules can be layered with a lipid hydrocolloid film coating through lamination to enhance their relatively low mechanical strength. Alternatively, they can be mixed with hydrophilic materials to form an emulsion complex. Despite having satisfactory mechanical properties and simple structures for their production and use, emulsion films are less effective than laminated films due to inadequate lipid dispersion. The complexity of producing multilayer films increases with the number of coatings used. Edible shellac has been used to coat fresh products and confections [74]. 

#### 4.1.5. Antioxidant-Based Edible Films

Developing edible packaging enriched with natural antioxidants is gaining significant attention [83,84]. This innovative approach enhances the cosmeceutical attributes of products, extends their longevity via minimizing weight reduction, and ensures the preservation of their freshness and sensory characteristics. It also safeguards the products from changes induced through light exposure, provides adaptability, and protects against moisture [85]. The efficacy of antioxidants as a packaging component is contingent on an appropriate choice of antioxidant (i.e., concentration and stability), the formulation of the packaging, the conditions under which it is stored, and the attributes of the end product. It is crucial to consider the properties of antioxidants during the packaging formulation process, particularly their miscibility, interaction, and solid and off-putting sensory characteristics (such as color, taste, and smell) that negatively impact a consumer’s acceptance of the product [86]. When incorporating antioxidants into edible films, it is vital to highlight the preference for naturally derived antioxidants. Adherence to governing bodies’ regulations and each country’s safety/quality standards is crucial, especially considering that edible packaging forms an essential component of the consumable portion (Table 7) [87].

Chitosan edible films are a superior packaging option for cosmetics. They have non-toxic nature, biodegradability, ability as a thickening agent, and antifungal, antioxidant, and moisturizing properties. This biomaterial can scavenge free radicals and chelate metal ions, thereby extending the shelf life of cosmetics [88,89]. 

The potential of edible packages can be enhanced through combining chitosan with essential oils (containing cinnamaldehyde, eugenol, rosemary, and thymol) [90,91], glutathione, vitamin E, vitamin C, α-carotene, phenolic acids, flavonoids, antioxidant biopeptides, and selenium, which can impart antioxidant properties to the films [3,92].

**Table 7:** Some patents in which antioxidants have been used to prepare edible films.

Patent Application	Patent Number	Plant-Based Ingredients	Details of the Invention	References
Taste-masking compositions and edible forms thereof	CA2834231A1	Chitosan, carboxymethylcellulose, methylcellulose, hydroxypropylmethylcellulose, locust bean gum, guar gum, carrageenan, xanthum gum, pullulan, pectins, and gum arabic	This patent illustrates edible films that mask the taste of products (cosmetics).	[93]
Methods and compositions for the treatment of skin	US9499419B2	Chitosan and chitin	This patent concerns formulations designed to treat and improve skin conditions such as acne, rosacea, and wrinkles. Various factors, including photodamage, aging, hormonal imbalances, hyper-pigmentation, melasma, and keratosis, can cause these conditions.	[94]

#### 4.1.6. Edible Sachets or Pouches

Edible sachets or pouches made from edible films or coatings can be used for samples or single-use portions of moisturizers, hair masks, or facial cleansers. Consumers can tear open the sachet, apply the product, and dispose of the packaging by eating or composting it. 

Notpla, a startup focused on sustainability, has introduced a novel packaging solution made from seaweed and plants to replace traditional plastic. This packaging was created by the branding agency Superunion. The material used by Notpla naturally decomposes in four to six weeks. It has been utilized in many ways, including thin films, coatings for takeaway containers, and condiment packets [95]. 

Xampla is a startup originating from the University of Cambridge. The startup offers high-quality plant-protein packaging (i.e., edible sachets, microcapsules, and coatings) that can prolong the shelf life of products [96]. 

## 5. Edible Cosmetics and Packaging Safety

Using bio-based packaging derived from organic waste can significantly reduce the cost of production of edible films. Furthermore, the widespread availability of such plant waste materials enhances their commercial viability due to the ease of sourcing. This innovative use of organic waste helps reduce waste and contributes to more sustainable and efficient packaging solutions. 

Edible films and coatings are classified as food ingredients, additives, contact substances, or packaging materials. Therefore, the components utilized in their production must be recognized as generally safe (GRAS) according to the Food and Drug Administration (FDA) guidelines. The colors, antioxidants, antimicrobials, and nutrients used to improve their performance must be identified and labeled on the packaging with their functional category and either their name or E-number [73]. A problem is the possible presence of allergens. It is common for polysaccharide extracts to contain protein residues. For example, guar gum extract, which contains less than 10% protein, can occasionally cause asthma and allergies [97,98]. However, due to the small number of reported cases, guar gum is not considered a significant food allergen [99]. Protein-based edible films and coatings can contain proteins rich in allergens, such as those found in milk (casein, whey), wheat (gluten), soy, and peanuts [100]. 

The safety of edible films is significantly influenced by the methods of modification and the choice of ingredients, yet these factors are often overlooked in edible film research. Certain risks are associated with creating harmful substances during film formation. The use of different cross-linking agents to enhance film properties can indeed lead to the formation of toxic materials, especially when they interact with gastrointestinal substances. For example, reduced graphene oxide showed lower toxicity than graphene oxide when used in a methylcellulose-based film [101]. 

Furthermore, incorporating nanomaterials into the film can produce various toxic effects on the human body depending on their chemical composition, particle size distribution, particle shape, and surface condition. The potential to cause oxidative stress and, in some cases, inflammatory responses or genotoxic effects are the most common effects observed in experimental studies. The intensity of the harmful effects depends on the nanomaterial dose. Their ability to penetrate human cells varies based on size. For example, 100 nm particles can easily penetrate cells, 40 nm particles can enter nuclei, and those below 35 nm can cross the blood–brain barrier. Moreover, smaller-sized particles can have more catalytic ability, and their reactive oxygen species-producing potential, adsorption rate, and binding capability can be comparatively higher than bigger-sized particles [84,102]. 

## 6. The Challenges to Enable the Development and Commercialization of Edible Packaging Materials

Edible packaging presents a unique solution to reduce waste and potentially provide consumers with added value and nutrition. However, the development and commercialization of edible packaging materials come with several challenges that need to be tackled by researchers, entrepreneurs, and regulatory bodies. 

Edible packaging materials must undergo safety testing that guarantees the absence of harmful substances or contaminants and be appropriately labeled to ensure that allergic or intolerant people do not encounter health risks. For example, edible films derived from seaweed or algae may contain high levels of iodine, which is harmful to people with thyroid conditions. Similarly, milk protein-based edible coatings can cause allergic reactions in some consumers. The functional properties and sensory attributes of edible packaging materials must be optimized to prevent moisture and nutrient loss, protect the cosmetic from microbial spoilage and oxidation, and not alter the cosmetic’s taste, texture, or appearance. For example, edible films made from fruit or vegetable purees can transfer their flavor or color to the food, which might not be desirable for some consumers [99]. The edible packaging materials may need to be reinforced or modified with cross-linking agents, plasticizers, or antimicrobial agents to enhance their stability under varying temperatures, humidities, and pressures. For instance, edible films made from starch or cellulose can be brittle and fragile and degrade quickly in moist or humid environments [103].

Similarly, edible coatings made from proteins or polysaccharides could be prone to enzymatic or microbial hydrolysis [104]. Sothornvit and Krochta conducted a study to examine the impact of enzymatic hydrolysis on the mechanical attributes and oxygen permeability of protein coatings. Their findings revealed that films formed from enzymatically hydrolyzed whey proteins exhibited enhanced flexibility compared with those made from non-hydrolyzed proteins, without any change in oxygen permeability [105]. In a separate experiment, Schmid, Hinz, Wild, and Noller created films using a fixed quantity of plasticizer and varying levels of hydrolyzed whey protein [106]. Their results demonstrated that an increased concentration of hydrolyzed whey protein significantly altered the films’ mechanical characteristics while the permeability to oxygen and water vapor remained constant. 

Finally, edible films should not significantly increase the production costs or retail prices of cosmetics. They should be compatible with simple and efficient processing techniques. Edible films made from chitosan or gelatin are expensive, complicated to source, and require complex and energy-intensive processing methods [107], and edible coatings made from wax or resin are incompatible with existing packaging equipment and systems [108,109]. 

## 7. Conclusions

Edible beauty represents an ideal blend of natural elements and scientific advancements. It offers a holistic approach to skincare, advocates for environmental preservation, and potentially provides added value and nutrition for consumers. As health, wellness, and sustainability values gain prominence among consumers, the demand for organic cosmetics is predicted to surge. This trend is expected to stimulate innovation and drive a beneficial shift in the beauty industry. Materials such as polysaccharides, proteins, lipids, and antioxidants can be obtained from food waste and processed into various mixtures or composites to meet the requirements of edible cosmetics and edible cosmetics packaging materials. These alternative materials offer advantages like easy accessibility and potential extraction from renewable resources at a relatively low cost. Significant advancements in active and intelligent packaging have been made through incorporating natural-based additives to enhance plasticizing and stabilizing properties and antibacterial and antioxidant functions. However, a joint effort between cosmetic industries, packaging producers, and research organizations is crucial for propelling innovation in edible beauty technology. The challenges to overcome include the shelf life of the edible packaging as well as the management of organoleptic characteristics that may not be attractive to consumers. Finally, regulatory authorities should improve guidelines regarding ingredient safety, labeling requirements, and good manufacturing practices for cosmetics and edible packaging to ensure the safety and effectiveness of cosmetic products and the interests of consumers.

## Figures and Tables

**Table 1:** Polymer-based products that are available commercially.

Commercial Name	Base Polymers	Application	References
Solagum AX^®^	Xanthan/acacia gum	Thickening–stabilizing–texturizing agent in sun care, face care, body care, hair care, make-up, hygiene (facial cleanser, shampoo, intimate hygiene, shower gel, etc.).	[18]
Syner-GX^®^	Xanthan/guar gum	Thickening–stabilizing–texturizing agent in cosmetic and personal care.	[19]
Nanoderm™	Bacterial cellulose	Wound care applications, skin tissue regeneration.	[20]
Chitoglycan^®^ PF	Carboxymethyl chitosan	Antimicrobial agent, bacteriostatic agent, film former, gel former in hair care, oral care, skin care.	[21]
Cellosize^TM^ Texture F4M	Hydroxypropypmethyl cellulose	Rheology modifier, binder, film former, and water-retention agent in hair care, color cosmetics, skincare.	[22]

**Table 2:** Some biopolymers used in cosmetic patents.

Patent Application	Patent Number and Publication Date	Edible Polymer	Details of the Invention	References
Hair cosmetic compositions containing sugar alcohol, saccharide compound, and pectin and methods of use.	WO2021173399	Pectin	Pectin provided hydration and moisture with a soft touch feel. Using pectin allowed the composition to achieve a stable viscosity and spray fluidity, which helped with batch scale-ups.	[23]
Cosmetic composition based on vegetable products.	CN113795241A 2019	Pectin	Texturing agent.	[24]
A film-forming composition comprising pectin.	WO2023036581A1	Pectin	The invention involved film compositions containing pectin, at least one additional film-forming polymer, and a setting system for use in pharmaceutical, veterinary, food, cosmetic, or other products like films for wrapping food, aspics, or jellies for predosed formulations like hard capsules.	[25]
Hydrophobically modified chitosan for use in cosmetics and personal care applications.	US20190159992A1	Chitosan	Cosmetic or a cosmetic application that included a hydrophobically modified biopolymer. The cosmetic application was selected from a group consisting of mascara, moisturizing creams, moisturizing lotions, facial cleansers, wrinkle-reducing gels/creams/lotions, shampoos, conditioners, soaps, deodorants, acne treatment, skin treatment, blemish concealers, coloring make-up, and controlled molecular release matrices for fragrances.	[26]
Cosmetic composition with casein.	KR20160026349A	Casein	The cosmetic composition using casein had a uniform coating surface, excellent maintenance, and a low eye-stimulation level.	[27]
Pectin fiber facial mask.	CN202933242U	Wood pulp	The utility model disclosed a pectin fiber facial mask completely formed of wood pulp fibers.	[28]
Mask sheet for improving skin elasticity and whitening.	KR20220052037A	Cellulose	The invention used a mast sheet containing albumin to enhance skin elasticity and shrink pores.	[29]
Cosmetic bio-cellulose sheet comprising an extract of puer tea and the preparation method thereof,	KR20180026923A	Cellulose	A biocellulose sheet for mask packs that contained a puer tea extract was the subject of the invention.	[30]

**Table 3:** Some patents in which biopolymers are used to prepare edible capsules or pods.

Patent Application	Patent Number and Publication Date	Edible Polymer	Details of the Invention	References
Edible bioplastic from seaweed and the manufacturing technology thereof	WO2014108887A2	Edible seaweed bioplastic	This invention relates to a plastic-like material that can be directly consumed, and the manufacturing technology uses seaweed as raw material.	[31]
Dissolvable and edible pods	US20180057230A1	Gelatin or other non-food-based materials, such as various polymers	The pod includes a dissolvable film of food-grade ingredients that dissolves quickly in hot or cold water. When the film from the pod dissolves in the water, the supplement is free to mix with the water to create a nutritional beverage designed for post-workouts. The pod is formed with a lower panel, forming a cavity for receiving the powder and covered with an upper panel.	[32]
Multi-compartment water-soluble capsules	WO2014202412A1	Polyvinyl alcohol	This patent refers to multi-compartment water-soluble capsules comprising at least two compartments made from water-soluble film, each containing part of a detergent composition. The capsules are employed in lotions, creams, and serums through dissolving them in water or other liquid.	[33]

**Table 4:** Some patents in which rice flour or vegetable starch are used in natural foundation, eyeshadow, or blusher.

Patent Application	Patent Number	Edible Polymer	Details of the Invention	References
Production method and product of rice starch hollow capsule.	CN106420657A	Rice starch	The invention explains how to produce hollow capsules made of rice starch.	[34]
A kind of blusher formula	CN108371649A	Starches	The invention discusses a cosmetic composition that utilizes insoluble native starch particles with specific characteristics. These starch particles have a smooth surface, free from roughness or edges.	[35]
Composition of cosmetic pressed powder modified with rice flour and lotus root starch and the preparation method thereof	CN111437233A	Rice flour	This patent describes the composition of a cosmetic pressed powder modified with rice flour and lotus root starch.	[36]
Color cosmetics using naturally derived materials	WO2009117625A2	Rice starch	The invention provides cosmetic compositions formulated with natural film-forming ingredients.	[37]

**Table 5:** Some patents in which plant-based ingredients are used to prepare edible lip balms or lipsticks.

Patent Application	Patent Number	Plant-Based Ingredients	The Invention Concern	References
Stick lip balm	EP2088995A1	Carnauba and candelilla waxes, jojoba esters, botanical butters; and non-jojoba ester waxes.	This patent illustrates a stick lip balm comprising at least 90% botanically derived materials.	[38]
Botanical butter stick lip balm	US7695727B2	Carnauba and candelilla waxes, jojoba esters, botanical butters; and non-jojoba ester waxes.	This patent illustrates a stick lip balm comprising at least 90% botanically derived materials.	[39]
Edible wolfberry lipstick and preparation method thereof	CN113876654A	Beta-cyclodextrin and starch	The invention is classified as a daily cosmetic and pertains to an edible wolfberry lipstick and its preparation method.	[40]

**Table 6:** Some patents in which biopolymers have been used to prepare edible film.

Patent Application	Patent Number	Edible Polymer	Details of the Invention	References
Sealed, edible film strip packets and methods of making and using them	KR101388955B1	Carboxymethyl cellulose and hydroxypropylmethyl cellulose (HPMC)	The packet is made with an edible film and contains a core composition. The design of the packet or sachet allows it to be placed in the mouth, where the film dissolves, releasing the core composition. In the most favored versions, the core composition includes a sugar alcohol like xylitol, which produces a cooling effect.	[75]
Edible products containing reconstituted plant ingredients	JP6963895B2	Alginate, pectin, soluble fiber	This invention is associated with products derived from plant fibers and consumable items that are enhanced with the application of extracts from plants. Essentially, it involves the creation of goods using plant fibers that are intended for consumption, and these edible goods are further enriched with the use of plant-based extracts. These extracts could potentially add additional flavors or health benefits to the products.	[76]
Films and drug delivery systems made from them	US20200146997A1	Polyethylene oxide and hydrophilic cellulosic polymers	Consumable multilayered film is applied to a body’s mucosal membrane. It is designed as a film of dosage units, each containing a specific amount of at least one active ingredient.	[77]
An edible collagen membrane and the preparation method thereof	CN105769659A	Collagen	The edible collagen membrane is oral edible. It can be used as a facial mask, pouch film, eye mask, cervacoria, hand film, pin film, etc.	[78]
Recombinant gelatins	US7393928B2	Gelatin	This invention concerns recombinant gelatins, their compositions, and their production and application processes.	[79]
Dissolvable film	US20070042023A1	Amylose starch, pectin, dextrin, chitosan, chitin, elsinan, levan, gelatin, collagen, zein, soy protein isolate gluten, whey protein isolate, casein, pyrrolidone, sodium, polyvinyl alginate	A film that exhibits instant wettability and rapid dissolution/disintegration projected to deliver or administer cosmetic or therapeutic substances.	[80]
Uniform film for fast-dissolving dosage forms containing taste-masking composition	JP5144776B2	Waxes as taste-masking agents	An edible film drug delivery that can contain cosmetics’ bioactive compounds.	[81]
Methods for marketing and generating revenue from edible thin films	WO2004045306A2	Wax-based edible film	The method helps prepare edible thin films for cosmetics.	[82]
